# Fecal Microbiota Restoration Modulates the Microbiome in Inflammation-Driven Colorectal Cancer

**DOI:** 10.3390/cancers15082260

**Published:** 2023-04-12

**Authors:** Travis J Gates, Ce Yuan, Mihir Shetty, Thomas Kaiser, Andrew C Nelson, Aastha Chauhan, Timothy K Starr, Christopher Staley, Subbaya Subramanian

**Affiliations:** 1 Department of Molecular Pharmacology and Therapeutics, University of Minnesota Medical School, Minneapolis, MN 55455, USA; 2 Department of Surgery, University of Minnesota Medical School, Minneapolis, MN 55455, USA; 3 Department of Obstetrics, Gynecology and Women’s Health, University of Minnesota Medical School, Minneapolis, MN 55455, USA; 4 Department of Laboratory Medicine and Pathology, University of Minnesota Medical School, Minneapolis, MN 55455, USA; 5 Masonic Cancer Center, University of Minnesota Medical School, Minneapolis, MN 55455, USA; 6 Center for Immunology, University of Minnesota Medical School, Minneapolis, MN 55455, USA

**Keywords:** microbiome, horizontal microbiota transfer, colitis, colorectal cancer, longitudinal sampling

## Abstract

**Simple Summary:**

Inflammation of the colon (colitis) can increase the risk of developing colorectal cancers. Gut microbiota may play a role in the development of this cancer. One potential way to prevent this cancer is to manipulate the community of bacteria in the colon. In a study using mice, we found that transferring the bacteria from the bedding of healthy mice to those with colitis increased the presence of *Akkermansia*, which may help reduce inflammation in the colon. Meanwhile, untreated mice had an increase in other types of bacteria, such as *Anaeroplasma* and *Alistipes*, which may contribute to inflammation. These findings suggest that certain types of bacteria may help reduce inflammation in the colon and prevent cancer.

**Abstract:**

Chronic inflammation of the colon (colitis) is a known risk factor for inflammatory-driven colorectal cancers (id-CRCs), and intestinal microbiota has been implicated in the etiology of id-CRCs. Manipulation of the microbiome is a clinically viable therapeutic approach to limiting id-CRCs. To understand the microbiome changes that occur over time in id-CRCs, we used a mouse model of id-CRCs with the treatment of azoxymethane (AOM) and dextran sodium sulfate (DSS) and measured the microbiome over time. We included cohorts where the microbiome was restored using cage bedding swapping and where the microbiome was depleted using antibiotics to compare to untreated animals. We identified consistent increases in *Akkermansia* in mice receiving horizontal microbiome transfer (HMT) via cage bedding swapping, while the control cohort had consistent longitudinal increases in *Anaeroplasma* and *Alistipes.* Additionally, fecal lipocalin-2 (Lcn-2), a marker of intestinal inflammation, was elevated in unrestored animals compared to restored and antibiotic-treated counterparts following HMT. These observations suggest a potential role for *Akkermansia, Anaeroplasma, and Alistipes* in regulating colonic inflammation in id-CRCs.

## 1. Importance

Preclinical models of inflammation-driven colorectal cancer (id-CRC) have shown that the microbiome regulates intestinal inflammation. However, most of these studies have relied on short periods of longitudinal microbiome sampling and a single mouse strain to conclude bacterial genera influencing id-CRC pathology. Here we utilized a mouse model of id-CRC. We show that horizontal microbiome transfer (HMT) through fecal restoration with healthy animals can longitudinally alter the microbiome of mice from Balb/c and C57BL/6 backgrounds compared to untreated controls. The longitudinal compositional changes showed consistent enrichments of *Akkermansia* in HMT-treated animals and *Anaeroplasma* and *Alistipes* in untreated controls. Additionally, we show that HMT can transiently decrease the concentration of inflammatory stool marker lipocalin-2 (Lcn-2) compared to untreated controls. Further investigation is warranted through additional preclinical and clinical investigations into how *Akkermansia, Alistipes,* and *Anaeroplasma* potentially alter epithelial inflammation and how they can be manipulated.

## 2. Introduction

Colorectal cancer (CRC) is the second leading cause of cancer-related death worldwide [1]. Inflammation of the colon has been shown to contribute to the development of CRC through various mechanisms, such as changing the local and systemic cytokine milieu through alteration of NF-kB and STAT3 signaling pathways promoting growth and survival [2,3]. Additionally, colitis increases the likelihood of induced gene mutations of the colonic epithelium and epigenetic alterations, providing conditions for growth [4,5,6]. Clinical observation also suggests a correlation between colonic neoplasia and increased mucosal prostaglandin, implicating a link between inflammation and CRC progression. Inflammatory- or colitis-driven CRC presents a unique microenvironment in which microbial dysbiosis has been implicated in driving or supporting tumor initiation and progression [7,8,9]. Dysbiosis of the microbiota can be loosely defined by a reduction in total microbial diversity and reduced abundances of commensal bacteria such as *Firmicutes* and *Bacteroidetes.* These commensal bacteria can provide the host with short-chain fatty acids such as butyrate, which has been shown to suppress colonic inflammation [10,11]. This is coupled with the increased abundance of pathobionts, such as members of the family *Enterobacteriaceae* [12,13]. However, these studies have yet to reach a consensus.

Many factors complicate determining causative associations between the microbiome and CRC pathogenesis. These factors include environmental, inter-individual, and dietary differences, variable experimental conditions (such as sampling parameters, i.e., stool or mucosa), longitudinal fluctuations, and lack of batch-to-batch reproducibility [14,15,16]. The advantages of using experimental mouse models include their homogenous genetic background, consistent environment and diet, and ability to perform longitudinal sampling, which can be temporally controlled. The most widely used model to induce colonic inflammation consists of administering a single intraperitoneal (IP) injection of azoxymethane (AOM), which is a carcinogen that causes DNA damage. This is followed by dextran sodium sulfate (DSS) administration via drinking water, which induces colonic inflammation [17]. This AOM/DSS model induces epithelial injury and promotes CRC development, mimicking human id-CRC [18].

Some studies have reported microbial composition and diversity alterations following AOM/DSS induction [19,20,21]. Others have shown that unique fecal compositions can be transplanted into germ-free mice and are associated with different tumor outcomes through the alteration of T-cell responses in AOM/DSS-induced models [22]. Fecal microbiota transplantation (FMT) of a CRC patient’s stool into *APC^min/+^* mice was also shown to lead to the accumulation of more intestinal tumors compared to healthy donor controls, suggesting a role for the tumor microbiome in CRC progression [23]. Restoration of fecal microbiota via FMT is an interesting approach to understanding microbial ecology. It is being explored in many colon diseases, including recurrent *Clostridiodes difficile* infection [24], irritable bowel disease, [25,26] and CRC [27]. Further, it has been shown that cohousing of animals can modulate the microbiome between mice through horizontal microbiome transfer (HMT) [28].

The longitudinal effects on the gut microbiota following HMT in AOM/DSS models of CRC have not been explored. In this study, we longitudinally analyzed the fecal microbiome of C57BL/6 and BALB/c mice that underwent swapping of used bedding from untreated mice to achieve HMT. In one group of mice, we homogenized the microbiome by swapping in bedding from untreated mice (restored microbiome) and compared it to mice that did not have their bedding swapped (unrestored microbiome). We observed significant and different early to late changes between restored and unrestored groups, consistent between C57BL/6 and BALB/c species following AOM/DSS administration.

## 3. Materials and Methods

### 3.1. Mice and Animal Husbandry

Three to four-week-old female C57BL/6 and BALB/c mice purchased from Jackson Laboratory were used in this study. On arrival at the animal facility, mice were randomly assigned to restored or unrestored groups (*n* = 5/group) and received an ear punch. Bedding on days 1–3 was pooled between experimental groups to normalize the baseline microbiome between restored and unrestored groups. Following baseline normalization, mice underwent AOM/DSS treatment, and stool samples were longitudinally collected 3x/week for 18 weeks. In week 18, mice were sacrificed and dissected. Mouse colons were fixed in 10% neutral buffered formalin and processed for histological analysis. All mice were housed in specific pathogen-free conditions in fully autoclaved cages for the duration of the experiment. The Institutional Animal Care and Use Committee approved all animal studies.

### 3.2. AOM/DSS Tumor Induction

Depending on the experimental design, female 3–4-week-old BALB/c or C57BL/6 mice were injected intraperitoneally with 10 mg azoxymethane (Sigma) per kg mouse weight. After five days, mice were treated with two cycles (1 week/cycle) of 2% dextran sodium sulfate (MP Biomedicals M.W. = 36,000–50,000) for five days through their drinking water, followed by a supply of regular water for the duration of the experiment. Mice were sacrificed in week 18 following AOM induction. Depending on the experimental setup, the antibiotics cocktail of 0.05 g/L of vancomycin (Boynton Health), 0.025 g/L of metronidazole (Boynton Health), and 0.2 g/L of streptomycin (Sigma) was administered in the drinking water of mice following the second cycle of DSS administration. During necropsy, colons were dissected and flushed with PBS to visualize tumors in the colon. Following visualization of tumors, tumor tissues were fixed in 10% neutral buffered formalin and sectioned.

### 3.3. Fecal Restoration and Stool Sample Collection

Mouse stool samples were longitudinally collected 3 times weekly for 18 weeks. Stool sample collection was conducted as follows: Mice were separated into individual autoclaved SPF cages lacking food and bedding and left for 1 h. On average, 3–5 fecal pellets were collected per mouse. Depending on group assignment, mice were removed from individual cages and returned to their respective cages. Stool samples were pooled by experimental groups and stored at −80 °C until processing for DNA isolation. During weeks 8, 9, and 10, the bedding from normal mice without AOM/DSS was distributed to cages for fecal restoration by HMT.

### 3.4. DNA Isolation and 16S rRNA Amplicon Sequencing and Analysis

Mouse fecal pellets of approximately 0.1 g were homogenized and processed using the DNA PowerSoil kit (Qiagen) with the QIACube automated platform following the manufacturer’s inhibitor removal technology (IRT) protocol. The V5-V6 hypervariable regions were amplified and sequenced using the BSF784/1064R primer set [29]. Sterile water and no template controls were carried through amplification and sequencing and did not produce amplicons. Samples underwent paired-end sequencing on a single run of a MiSeq platform (Illumina, Inc., San Diego, CA, USA) at a length of 301 nucleotides (nt). All sequence processing was performed using Mothur software version 1.35.1 [30]. Raw fastq files were trimmed to 150 nt sequences to remove low-quality regions and were paired-end merged using the fastq-join function [31]. Reads were further trimmed to remove reads with mean quality scores < 35 over a 50 nt window, homopolymers > 8 nt, ambiguous bases, and more that 2 nt mismatches to primer sequences. High-quality sequences were aligned to the SILVA database (version 132) [32] and subjected to a 2% pre-clustering step to remove likely errors [33]. Chimeric sequences were identified and removed using the UCHIME package [34]. Operational Taxonomic Units (OTUs) were assigned at 99% similarity using the complete-linkage clustering algorithm, and taxonomy was assigned using version 16 of the Ribosomal Database Project [35]. The raw fastq files are available at https://www.ncbi.nlm.nih.gov/sra/PRJNA915052.

### 3.5. Histopathological Analysis

Mouse colons were fixed overnight in 10% neutral buffered formalin at the experimental endpoint. Following fixation, solutions were changed to 70% ethanol before submission to the clinical and translational sciences institute at the University of Minnesota. Tissues were paraffin-embedded, and two sections per mouse were stained with hematoxylin and eosin. Board-certified pathologists reviewed tissue sections for dysplasia and acute and chronic inflammation.

### 3.6. Quantification of Fecal Lipocalin-2 (Lcn-2) by ELISA

Initially, 0.2 g of frozen fecal samples was reconstituted in PBS with 0.1% Tween 20 (100 mg/mL). Subsequently, 3 mm glass beads were added to the mixture and vortexed 3 times in 30 s bursts to obtain a homogenous fecal suspension. The samples were then centrifuged for 10 min at 12,000 rpm at 4 °C. Clear supernatants were collected and stored at −20 °C prior to analysis. Lcn-2 levels were approximated using a Duoset murine Lcn-2 ELISA kit (R&D Systems, Minneapolis, MN) following the manufacturer’s protocol. Four time points before and after fecal restoration (W2, W7, W10, W15) were selected for fecal Lcn-2 analysis. Samples for each experimental condition were run in triplicate. Calibration standard curves (500 pg/mL–7.81 pg/mL) were used to extrapolate Lcn-2 concentrations and averaged across experimental time points and conditions. Reagent blank 1.0% BSA in PBS was used as a negative control.

### 3.7. Statistics

Alpha diversity of microbial communities was assessed using Shannon and Chao1 indices. Bray–Curtis dissimilarity matrices [36] were calculated and used for ordination through principal coordinates analysis [37]. These matrices were also used to determine differences in beta diversity by analysis of similarity (ANOSIM) [38]. Bonferroni corrections for multiple comparisons were performed for ANOSIM analyses. The SplinectomeR R package [39] and its permuspliner functions were used to assess significant differences in the longitudinal abundances of bacterial genera between restored and unrestored groups using standard 999 permutations. One-way unpaired equal variance Student’s T-tests were conducted to assess statistically significant differences in concentrations of fecal Lcn-2. All statistics were evaluated at α = 0.05 unless otherwise corrected for multiple comparisons.

## 4. Results

### 4.1. Microbial Community Composition and Diversity between Restored and Unrestored Groups Are Longitudinally Different

**Figure 1:**
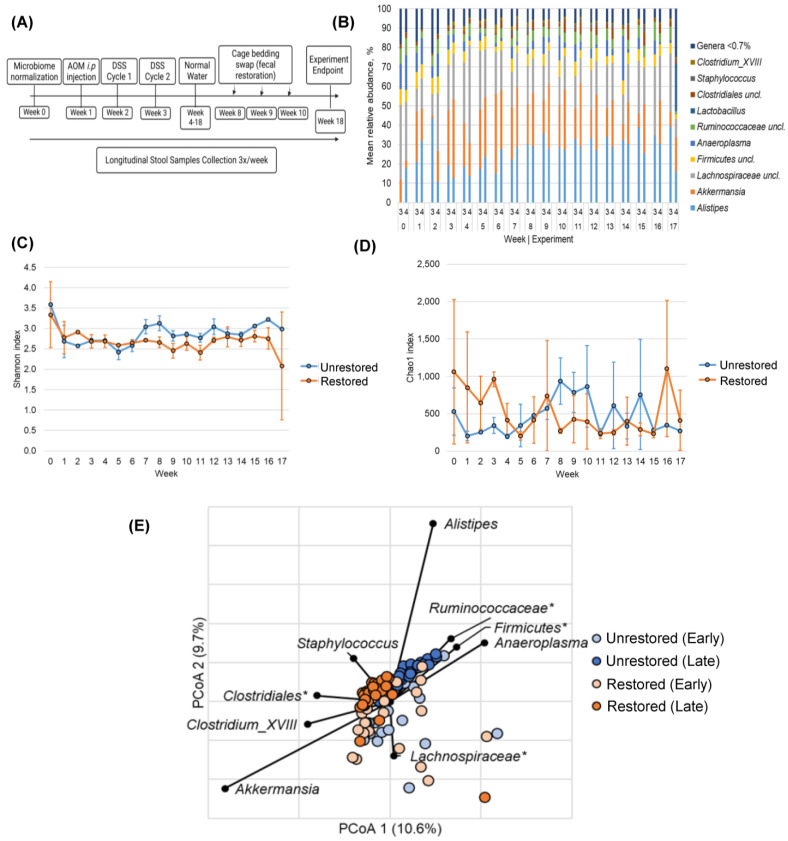
Microbiome composition and diversity in Balb/c mice. (**A**) Percentage of the relative abundance of bacterial genera in Balb/c mice following AOM/DSS administration between (3) unrestored and (4) restored groups. Genera that accounted for <0.7% of mean sequence reads were consolidated for clarity. (**B**) Longitudinal Shannon index between no restoration (blue) and restoration (orange). Error bars reflect standard deviation. (**C**) Longitudinal Chao1 index between no restoration (blue) and restoration (orange). (**D**) Principal coordinates analysis (**E**) Correlated taxa associated with ordination position on either axis (Spearman *p* > 0.05) are overlaid on the PCoA plot. (*) Indicates genera that could not be further classified.

We longitudinally compared the microbial community changes following AOM/DSS administration between unrestored (no HMT) and restored (HMT from normal Balb/c fecal bedding swap once weekly during weeks 8, 9, and 10) groups. Mouse fecal pellets were longitudinally collected three times weekly for 17 weeks from Balb/c mice in unrestored *n* = 5 and restored *n* = 5 groups (Figure 1A). The overall mean percentage of the relative abundance of bacterial genera in restored and unrestored groups was relatively stable and similar (Figure 1B). Longitudinally, the microbial communities in mice with restored microbiota had significantly lower Shannon index values than unrestored mice (*p* = 0.035; Figure 1C); however, no significant differences were observed in Chao1 (*p* = 0.084; Figure 1D).

Next, we sought to investigate the difference in community composition between restored and unrestored groups using Bray–Curtis dissimilarity. Samples in both treatment groups were considered as “early” (weeks 0–7; before microbiota restoration) and “late” (weeks 8–18; at the time of and following HMT). Principal coordinates analysis (PCoA) showed significant early and late changes in the microbiome of Balb/c mice treated with AOM/DSS (Figure 1E). The unrestored group had significant early and late changes (ANOSIM R = 0.499, *p* < 0.001) within the group (Figure 1E). Similarly, we also observed significant early and late changes in the restored group (ANOSIM R = 0.261, *p* < 0.001). Microbiota composition also differed significantly between experimental groups among the later “late” samples, but not samples collected prior to week 8 (R = 0.530 and 0.081, *p* < 0.001 and *p* = 0.01; Bonferroni-corrected α = 0.008; Figure 1E)

Spearman correlation was used to identify bacterial genera associated with the late differences in the microbial community between unrestored and restored groups (Figure 1E). *Akkermansia, Clostridium*_clade XVIII, and members of the order *Clostridiales* that could not be further classified were compositionally correlated with the restored group (Figure 1E). This was an interesting observation as *Akkermansia* and *Clostridium*_XVIII have been implicated as having probiotic properties and are associated with reduced intestinal inflammation through production of short-chain fatty acids such as butyrate [40,41].

In contrast, microbial communities in the restored group were correlated with greater relative abundances of the bacterial genera *Alistipes, Anaeroplasma, and Ruminococcaceae*, as shown in Figure 1E. We were unable to classify *Rumminococcaceae* further in our 16S rRNA analysis. Interestingly, *Ruminococcus gnavus* is associated with Crohn’s disease, likely through the ability of *R. gnavus* to synthesize and secrete glucorhamnan polysaccharides, which can lead to TNFα secretion by dendritic cells [42]. Additionally, the increased abundance of potentially pathogenic *Alistipes* is consistent with recent investigations of AOM/DSS-induced cancer [43]. These data suggest that HMT altered the microbial composition in the restored group compared to the unrestored group, potentially shifting toward a less inflammatory community composition.

### 4.2. Longitudinal Differences in the Microbiome between Restored and Unrestored Groups Is Significant

Next, we sought to investigate longitudinal changes in bacterial genera between restored and unrestored groups using the SplinectomeR permuspliner function, which can be used to assess longitudinal microbiome data [39]. Significant longitudinal differences were observed between groups among the genera *Alistipes* (*p* = 0.032), *Akkermansia* (*p* = 0.001), *Anaeroplasma* (*p* = 0.032), *Ruminococaceae* (further unclassified) (*p* = 0.001), *Clostridiales* (further unclassified) (*p* = 0.001), and *Clostridium_XVIII* (*p* = 0.001) (Supplemental Figure S1A–F). Significant longitudinal differences in the Shannon index (*p* = 0.035) between groups were also observed (Supplemental Figure S1G). Notably, the longitudinal significant difference between unrestored and restored is a somewhat conflicting result as previous work has shown that alpha diversity has decreased longitudinally in AOM/DSS models of intestinal inflammation.

The longitudinal abundance of *Akkermansia* was significantly different between unrestored and restored groups following fecal restoration (Supplemental Figure S1B). For *Akkermansia*, the mean relative abundance was significantly greater in the restored group compared to the unrestored in weeks 9–17. This was also reflected in our Bray–Curtis dissimilarity and Spearman correlation analysis in Figure 1E. Many bacterial genera identified from Spearman correlations were also longitudinally altered between unrestored and restored groups in SplinectomeR permuspliner analysis.

### 4.3. Intestinal Inflammation Marker Lipocalin-2 Is Decreased in Restored Balb/c Mice

**Figure 2:**
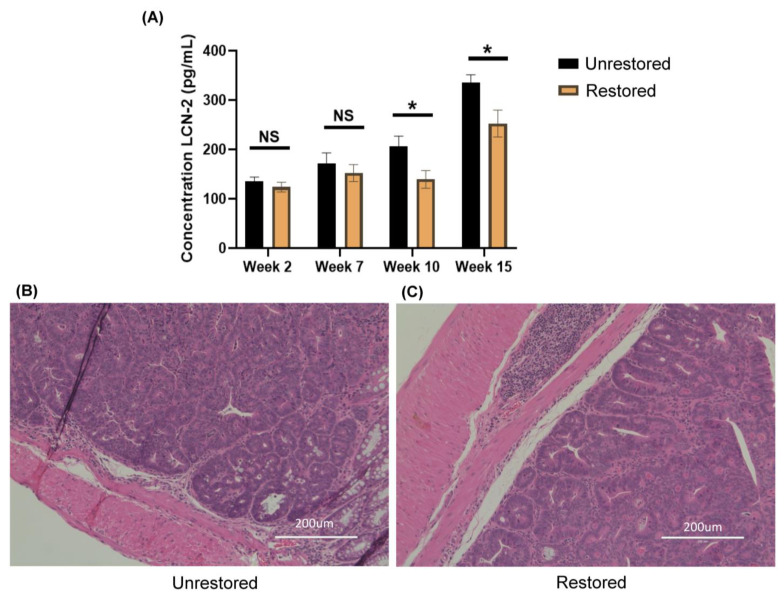
Fecal restoration alters colonic inflammation in Balb/c mice. (**A**) Fecal concentrations of Lcn-2 in unrestored (black) restored (orange) groups at weeks 2, 7, 10, and 15 (left to right) (* *p* < 0.05) (*n* = 3) (+/− SD). (**B**) Hematoxylin and eosin staining of the colon tissues of unrestored (**C**) Restored Balb/c mice.

To assess differences in intestinal inflammation before and after HMT in AOM/DSS-treated animals, we utilized a semi-quantitative sandwich ELISA (R&D systems, Minneapolis, MN) to probe fecal concentrations of lipocalin-2 (Lcn-2) [44,45]. Fecal Lcn-2 concentrations are a non-invasive marker of intestinal inflammation. Two time points were used before HMT (weeks 2 and 7), and two time points post-HMT (weeks 10 and 15) were selected to quantify fecal concentrations of Lcn-2 in restored and unrestored groups. In weeks 2 and 7, we did not observe significant differences in fecal Lcn-2 concentrations (week 2, 136.1 ± 8.0 pg/mL and 123.7 ± 9.8 pg/mL, *p* = 0.082, *n* = 3) (week 7, 171.1 ± 21.7 pg/mL and 154.2 ± 17.2 pg/mL, *p* = 0.154, *n* = 3) between unrestored and restored animals (Figure 2A). Post-HMT, we did observe significant decreases in fecal Lcn-2 concentrations (week 10, 206.1 ± 20.9 pg/mL and 139.4 ± 17.8 pg/mL, *p* = 0.007, *n* = 3) (Week 15, 335.4 ± 16.2 pg/mL and 252.4 ± 27.3 pg/mL, *p* = 0.005, *n* = 3) in unrestored and restored animals (Figure 2A). These data suggest that HMT could potentially reduce Lcn-2 concentrations and could have transiently reduced intestinal inflammation in restored animals compared to unrestored counterparts.

Despite significant differences in fecal Lcn-2 concentrations, we did not observe any pathological differences at the experiment endpoint when looking at the hematoxylin and eosin staining of fixed colon tissues (Figure 2B). Both restored and unrestored animals at the experiment endpoint presented histology ranging from high-grade dysplasia to high-grade intramucosal adenocarcinoma. Additionally, restored and unrestored animals presented with acute and chronic inflammation ranging from focally mild to focally moderate and focally marked. These observations also align with attempted colonoscopy of unrestored and restored animals in the early stage. However, a colonoscopy was impossible at late stages due to bloody stool and inflammation within the colon. Although there were no significant histological differences at the experiment endpoint, we observed significant differences post-HMT in fecal Lcn-2 concentrations. This observation led us to consider that the protective effects of HMT were likely transient, and more frequent treatment with HMT could impact pathology between groups.

### 4.4. Bacterial Community Compositions Are Similar between Balb/c and C57BL/6 in Late Restored Groups

We previously observed altered longitudinal fecal microbiomes of restored Balb/c mice compared to unrestored mice. Considering the variable fluctuations in microbiome analysis, we questioned whether our observations were specific to Balb/c. We used 3–4-week-old Balb/c and C57BL/6 mice in the AOM/DSS model to test this. Additionally, we included a group of 3–4-week-old C57BL/6 mice treated with an antibiotics cocktail of vancomycin (0.05 g/L), metronidazole (0.025 g/L), and streptomycin (0.2 g/L) to deplete mouse microbiomes as a control to ensure our longitudinal data acquisition and analysis pipelines were working appropriately. Interestingly, antibiotics-treated mice observed lower-grade dysplasia compared to unrestored and restored counterparts, which was in line with previous studies [20]. Fecal restoration via HMT was again performed during weeks 8, 9, and 10.

**Figure 3:**
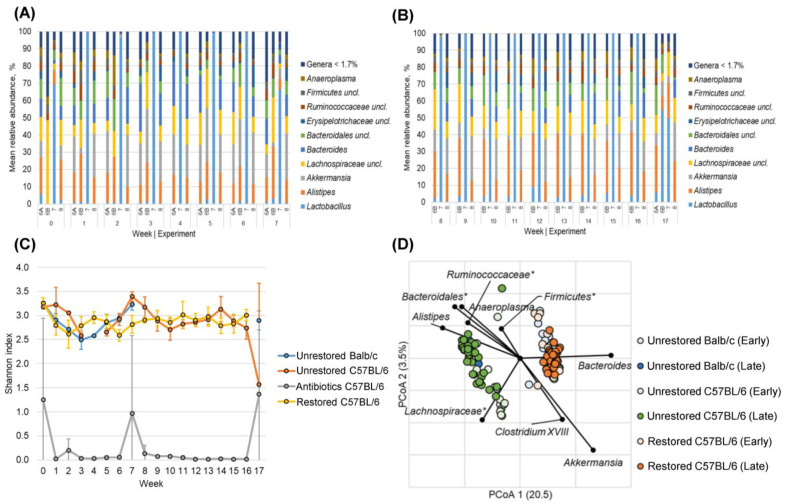
Microbiome composition and diversity in C57BL/6 mice. (**A**) Percentage of the relative abundance of bacterial genera (W0-W7) in EXP6A (Balb/c unrestored), EXP6B (C57BL/6 unrestored), EXP7 (C57BL/6 + ABX), and EXP8 (C57BL/6 C57BL/6 restored) following AOM/DSS administration. Genera that accounted for <0.7% of mean sequence reads were consolidated for clarity. (**B**) Percentage of the relative abundance of bacterial genera (W8-17). (**C**) Longitudinal average Shannon index, with groups shown in blue (unrestored Balb/c), orange (unrestored C57BL/6), yellow (restored C57BL/6), and gray (antibiotics C57BL/6). (**D**) Principal coordinates analysis with correlated taxa associated with ordination position on either axis (Spearman *p* > 0.05) are overlaid on the PCoA plot. (*) Indicates genera that could not be further classified.

Longitudinally stable mean relative abundances of genera between Group 6A (Balb/c), Group 6B (C57BL/6 unrestored), Group 7 (C57BL/6 +Antibiotics), and Group 8 (C57BL/6 restored) were observed (Figure 3A,B). These data from C57BL/6 were consistent with our previous observation in Balb/c mice. As expected, we observed depleted alpha diversity in Shannon and Chao1 indices for mice receiving antibiotics compared to restored and unrestored groups (Figure 3C and Supplemental Figure S2). Similar alpha diversity was observed between the restored and unrestored groups, which was not significant when compared using ANOSIM. Interestingly, we did not notice decreasing alpha diversity in restored C57BL/6 mice compared to unrestored mice, as was observed in Balb/c mice.

Next, we used Bray–Curtis dissimilarity and PCoA to compare restored and unrestored groups’ early and late microbial compositions. The early compositional signature between Balb/c and C57BL/6 unrestored animals differed significantly (ANOSIM R = 0.833, *p* < 0.001; Figure 3D). However, unrestored mice showed more similarity in late microbial compositions between Balb/c and C57BL/6 mice (ANOSIM R = −0.101, *p* = 0.723; Figure 3D). These observations suggested that even though C57BL/6 and Balb/c unrestored mice had significantly different initial microbial compositions after AOM/DSS treatment, similar compositions were observed between strains of mice when left untreated.

Furthermore, we also observed significant differences between late restored C57BL/6 and unrestored C57BL/6 mice (R = 0.897, *p* < 0.001; Figure 3D). Spearman correlation was used to investigate bacterial genera associated with differences in ordination position. Interestingly, we noticed similar taxa (i.e., *Bacteroides, Clostridium*_XVIII, and *Akkermansia)* were correlated with late compositions of the restored group (Figure 3D), similar to our prior observations in Balb/c mice. Additionally, unrestored C57BL/6 communities were associated with a greater relative abundance of *Anaeroplasma, Alistipes, Lachnospiraceae, Bacteroidales* (not further classified)*,* and *Ruminococcaceae* (not further classified). Notably, the genera *Ruminococcaceae, Alistipes,* and *Anaeroplasma* were found in greater abundance in late samples among untreated Balb/c and C57BL/6 mice. Similarly, *Akkermansia* and *Clostridium*_XVIII were found at greater relative abundances in the restored microbiota of both mouse genotypes.

### 4.5. Inflammatory and Pathological Signatures between Balb/c Mice and C57BL/6 Mice Are Different

**Figure 4:**
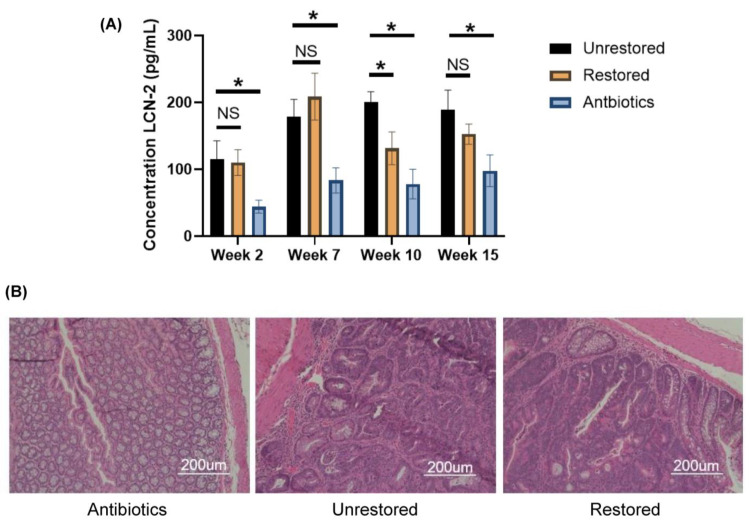
Fecal restoration modulates intestinal inflammation in C57BL/6 mice. (**A**) Fecal concentrations of Lcn-2 in unrestored (black), restored (orange), and antibiotics-treated mice (blue) in weeks 2, 7, 10, and 15 (left to right) (* *p* < 0.05) (*n* = 3) (+/− SD). (* *p* < 0.05) (NS *p* > 0.05) (**B**) Hematoxylin and eosin staining of the colon tissues of unrestored, antibiotics-treated, and restored C57BL/6 mice.

We again used ELISA to probe fecal concentrations of Lcn-2 pre-HMT (weeks 2 and 7) and post-HMT (weeks 10 and 15) in restored, unrestored, and antibiotics-treated animals to observe alterations in intestinal inflammation between groups. We did not observe significant differences in fecal Lcn-2 concentrations pre-HMT (week 2, 115.3 ± 27.4 pg/mL and 110.1 ± 19.3 pg/mL, *p* = 0.400, *n* = 3) (week 7, 178.6 ± 26.0 pg/mL and 208.6 ±35.1 pg/mL, *p* = 0.150, *n* = 3) between unrestored and restored animals (Figure 4A). We did observe significant differences in fecal Lcn-2 concentrations post-HMT (week 10, 200.8 ± 15.3 pg/mL and 131.4 pg/mL ± 24.5 pg/mL, *p* = 0.007, *n* = 3) between unrestored and restored groups (Figure 4A). This result was consistent with our previous investigation in Balb/c mice post-HMT. However, we did observe a difference in fecal concentration of Lcn-2 (188.8 ± 29.5 pg/mL and 152.5 ± 15.1 pg/mL, *p* = 0.065, *n* = 3) between unrestored and restored groups in week 15, but it did not reach statistical significance (*p* = 0.065). Interestingly, for all four time points, antibiotics-treated animals had statistically significantly lower concentrations of fecal Lcn-2 (44.2 ± 9.7 pg/mL, *p* = 0.003, *n* = 3) (83.5 ± 18.9 pg/mL, *p* = 0.003, *n* = 3) (78.1 ± 22.1 pg/mL, *p* = 0.024, *n* = 3) (97.9 ± 23.8 pg/mL, *p* = 0.014, *n* = 3) than both unrestored and restored animals (Figure 4A). This was in line with previous investigations in C57BL/6 animals treated with antibiotics in AOM/DSS models of intestinal inflammation. Additionally, in general, Balb/c mice presented with elevated levels of fecal Lcn-2 across all treatment conditions compared to C57BL/6 mice, which was also recapitulated in H and E stains of fixed colon tissues, as shown in Figure 2.

Even though we observed a statistically significant decrease in fecal Lcn-2 concentrations in week 10 following HMT, this did not influence the pathological observations at the experimental endpoint between restored and unrestored animals (Figure 4B). Mice in restored and unrestored groups at the experiment endpoint presented with high-grade dysplasia to high-grade intramucosal adenocarcinoma. Furthermore, both restored and unrestored animals presented with acute inflammation, ranging from minimal to focally moderate, and chronic inflammation, ranging from mild to focally moderate. These observations suggested that any beneficial effect from HMT, as determined by concentrations of fecal Lcn-2, was likely transient and did not influence overall pathological outcomes between unrestored and restored groups. However, mice receiving antibiotics were absent of dysplasia and showed only minimal acute inflammation and mild chronic inflammation. This was consistent with fecal Lcn-2 concentrations as antibiotics-treated animals had significantly reduced levels of Lcn-2 compared to restored and unrestored animals throughout all sampled time points. These data further support a role or niche of the gut microbiota in regulating intestinal inflammation as mice treated with antibiotics presented with less advanced-stage disease than unrestored and restored groups.

### 4.6. Alistipes, Akkermansia, and Anaeroplasma Are Longitudinally Altered in C57BL/6 Restored and C57BL/6 Unrestored Mice

**Figure 5:**
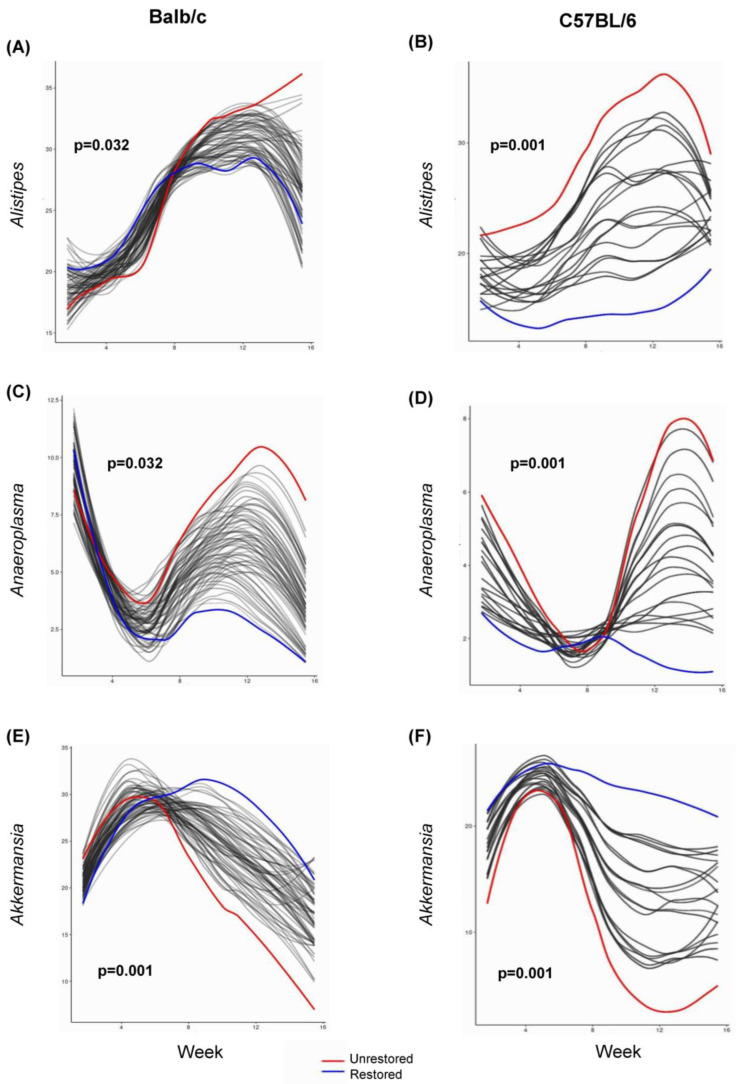
Longitudinally significant genera conserved between mouse genotypes. Longitudinal differences in the relative abundance of genera significantly differ between no restoration (red) and restoration (blue) groups as determined by SplinectomeR. (**A**) *Alistipes,* Balb/c (*p* = 0.032); (**B**) *Alistipes,* C57BL/6 (*p* = 0.001); (**C**) *Anaeroplasma,* Balb/c (*p* = 0.032); (**D**) *Anaeroplasma,* C57BL/6 (*p* = 0.001); (**E**) *Akkermansia,* Balb/c (*p* = 0.001); (**F**) *Akkermansia,* C57BL/6 (*p* = 0.001).

Next, we investigated longitudinal changes in bacterial genera between C57BL/6 restored and unrestored groups using the SplinectomeR permuspliner function [39]. We observed significant longitudinal differences in *Alistipes* (*p*= 0.001), *Akkermansia* (*p* = 0.001), *Bacteroides* (*p* = 0.001), *Erysipelotrichaeae* (*p* = 0.001), and *Anaeroplasma* (*p* = 0.001) between unrestored and restored groups (Figure 5 and Supplemental Figure S3). These bacterial genera were also identified in our Bray–Curtis dissimilarity analysis and were correlated using Spearman correlation. Additionally, we observed longitudinally significant differences in *Bacteroides* between unrestored C57BL/6 mice and restored C57BL/6 mice. However, this was not observed in Balb/c mice. *Compared to healthy controls, Bacteroides are commonly observed in patients with irritable bowel disease* [46,47]. When comparing Balb/c and C57BL/6 from all experiments, we consistently saw longitudinal increases in *Akkermansia* in the restored group compared to the unrestored, along with consistent observations of an increased abundance of *Alitipes* and *Anaeroplasma* in unrestored compared to restored Balb/c and C57BL/6 mice.

## 5. Discussion

Interactions between the microbiota that contribute to host intestinal inflammation are increasingly being investigated. Associations have been reported between bacterial genera and disease phenotypes in inflammatory bowel diseases and CRC [7,48,49]. Many variables render causative associations between the microbiome and disease pathology challenging [14,15,16]. Our study aimed to use an established mouse model of id-CRC [17,18] to longitudinally track shifts in the microbiome of AOM/DSS-treated mice and assess how fecal restoration influences longitudinal microbial signature and disease pathology. Our study demonstrated that longitudinal compositional changes over 17 weeks are similar between Balb/c and C57BL/6 mice following AOM/DSS treatments (Figure 1 and Figure 3). Additionally, we demonstrated that horizontal microbiome transfer (HMT) restoration from untreated mice through bedding swaps led to consistent longitudinal increases in *Akkermansia* in Balb/c and C57BL/6 mice compared to their respective unrestored controls, along with consistent observations of an increased abundance of *Anaeroplasma* and *Alistipes* in unrestored Balb/c and C57BL/6 mice.

Clinical investigations have reported that the relative abundance of *Akkermansia* is decreased in stool samples of patients with active ulcerative colitis compared to patients with quiescent ulcerative colitis and healthy controls [50]. We reason that observing consistent longitudinal increases in *Akkermansia* in restored mice compared to unrestored mice may represent a more quiescent phenotype following fecal restoration. Additionally, it has been reported that oral administration of *A. muciniphila* strain BAA-835 significantly ameliorated intestinal inflammation following DSS-induced inflammation and was dependent on NLRP3 expression [51]. Others have shown that *A. muciniphila* strain BAA-835 can reduce inflammatory cytokine expression and reduce mucosal inflammation in DSS models of inflammation [39,40]. Knowing that *Akkermansia* can reduce inflammatory cytokine expression could explain why we observed decreased fecal Lcn-2 concentrations in week 10 following HMT in both genotypes of mice (Figure 2 and Figure 4). Nevertheless, these temporal increases in *Akkermansia* in restored groups did not yield a difference in pathological outcomes between restored and unrestored groups, so this effect was likely transient. More frequent administration of HMT could potentially yield alterations in pathological outcomes; however, without further investigation, the overall impact of HMT on disease pathology remains unknown.

Additionally, mice treated with antibiotics observed less dysplasia and inflammation than restored and unrestored animals. Our observations corroborate previous investigations and suggest that microbiota regulates intestinal inflammation, which can be seen in mice lacking a microbiome exhibiting less aggressive disease [20]. Furthermore, our Lcn-2 data support different pathological outcomes in antibiotics-treated mice as, throughout all sampled time points observed, there were significantly lower concentrations of Lcn-2 compared to unrestored and restored animals (Figure 4). We also observed significantly lower concentrations of Lcn-2 in C57BL/6 mice compared to Balb/c mice across treatment conditions. This supports our observations from hematoxylin and eosin staining, where Balb/c mice tended to present more frequently with intramucosal adenocarcinoma and focally marked inflammation than C57BL/6 counterparts. In general, C57BL/6 mice presented with less aggressive disease than Balb/c mice. Our studies suggest that these observations should be considered in future investigations of id-CRCs, as choosing an appropriate model can influence pathological outcomes. Nevertheless, these observations suggest that fecal restoration could contribute to an altered colonic inflammatory response through longitudinal increases in *Akkermansia.*

We also observed significant longitudinal increases in *Alistipes* and *Anaeroplasma* in the unrestored compared to the restored group in Balb/c and C57BL/6 mice (Figure 1 and Figure 3 and Supplemental Figures S1 and S3). The observation of an increased abundance of potentially pathogenic *Alistipes* is consistent with recent investigations of AOM/DSS-induced CRCs [43]. Additionally, more mechanistic insight has been gained by studies showing that *Alistipes* abundance is sufficient to induce colitis in *IL10* ^-/-^ mice and can lead to tumorigenesis [52]. *Alistipes* has also been identified as a predominant genus of CRC patients from metagenome-wide association studies and is more abundant in carcinomas compared to adenomas and healthy controls [53]. Our observation of an increased relative abundance of *Alistipes* in unrestored groups compared to restored groups could reflect an increased abundance of *Alistipes* contributing to id-CRCs. Furthermore, *Alistipes* abundance has been implicated in the response to immune checkpoint inhibitors (ICIs) in preclinical models [54]. Iida et al. showed that antibiotic-treated animals bearing MC38 tumors observed resistance to anti-IL10 antibody treatment [55]. Additionally, *Alistipes* and *Ruminoccocus* were positively correlated with TNF-dependent anti-tumor immune activation in response to immunotherapy [55]. Routy et al. also implicated *Alistipes indistinctus* in response to ICIs and other commensal gut microbes in reversing resistance to ICIs [56]. Routy et al. administered *Alistipes indistinctus* to tumor-bearing animals and reversed resistance to ICIs [56]. Further studies are needed to fully illuminate how genera can modulate immune responses in the context of ICI resistance to enhance response rates in different malignancies so this knowledge can be translated into clinics.

We also observed consistent increases in the relative abundance of *Anaeroplasma* in unrestored compared to restored groups for both murine backgrounds. It has been reported that *Anaeroplasma* is enriched in mice receiving AOM/DSS compared to healthy controls, [21] and our work demonstrates a similar profile. However, conflicting results have also been reported. For instance, *Anaeroplasma* was shown to be decreased in the stool of mice with a common CRC driver gene *APC* mutation compared to the wild type [57]. However, different inflammatory niches between models could explain this observed difference in the abundance of *Anaeroplasma.* Further, it has been reported that *Anaeroplasma* strongly correlates with intestinal IgA and TGF-B secretion and regulates intestinal inflammation [58]. This is also conflicting as IgA increases in patients with IBD [59], while TGF-B is a known immunosuppressive cytokine [60].

Lastly, we also observed significant longitudinal increases in *Ruminococcaceae* in Balb/c restored mice compared to unrestored Balb/c but not C57BL/6 mice. Interestingly, *Ruminococcus gnavus* is associated with Crohn’s disease, likely through the ability of *R. gnavus* to synthesize and secrete glucorhamnan polysaccharides, which can lead to TNFα secretion by dendritic cells [42]; it is also enriched in CRC patients compared to normal controls [61]. We did not see this recapitulated in Balb/c and C57BL/6 mice. However, we reason this as evidence that fecal restoration longitudinally shifted the microbiome and modulated fecal Lcn-2 concentrations between restored and unrestored mice between genotypes.

## 6. Conclusions

This study investigated how HMT through cage bedding swapping with untreated animals could influence the microbiome and pathological outcomes of Balb/c and C57BL/6 mice treated with AOM/DSS to mimic id-CRC. We observed consistent and significant increases in *Akkermansia* in restored groups compared to unrestored counterparts in both genotypes. At the same time, we observed consistent increases in *Anaeroplasma* and *Alistipes* in unrestored animals compared to restored counterparts across genotypes. These observations were correlated with reduced fecal Lcn-2 concentrations post-HMT in restored mice compared to unrestored counterparts. We also observed differences in dysplasia and inflammatory signatures between Balb/c and C57BL/6 mice, which should be considered in future investigations.

In comparison, fecal restoration through HMT did not yield pathologically different outcomes between restored and unrestored animals. Temporal differences in fecal Lcn-2 concentrations were observed post-HMT. Future studies should seek to illuminate the direct microbiota–host mechanisms of *Akkermansia, Anaeroplasma,* and *Alistipes* for their roles in regulating intestinal inflammation and CRC development in different model systems.

## Supplementary Materials

The following supporting information can be downloaded at: https://www.mdpi.com/article/10.3390/cancers15082260/s1, Supplementary Figure S1: Longitudinally significant genera. Longitudinal differences in the relative abundance of genera significantly differ between no restoration (red) and restoration (blue) groups as determined by SplinectomeR. (A) Alistipes (*p*= 0.032); (B) Akkermansia (*p* = 0.001); (C) Anaeroplasma (*p* = 0.032); (D) Ruminococacea_uncl (*p* = 0.001); (E) Clostridiales_uncl (*p* = 0.001); (F) Clostridium_XVIII (*p* = 0.001); (G) Shannon index (*p* = 0.035). Supplemental Figure S2: Alpha diversity analysis: Longitudinal average Chao1 index between unrestored Balb/c (blue), unrestored C57BL/6 (orange), restored C57BL/6 (yellow), and antibiotics C57BL/6 (gray) groups. Supplemental Figure S3: Longitudinally significant genera: Longitudinal analysis between unrestored C57BL/6 (red) and restored C57BL/6 (blue) mice using SplinectomeR. (A) *Alistipes* (*p* = 0.001); (B) *Akkermansia* (*p* = 0.001); (C) *Bacteroides* (*p* = 0.001); (D) *Erysipelotrichaeae* (*p* = 0.001); (E) *Anaeroplasma* (*p* = 0.001).
