# Supplementary material for: Fecal Microbiota Restoration Modulates the Microbiome in Inflammation-Driven Colorectal Cancer
Source: Cancers (Basel). 2023 Apr 12;15(8):2260. doi: 10.3390/cancers15082260 (PMC10137216; doi:10.3390/cancers15082260)

Supplemental Figures

Supplementary Figure S1A

A)

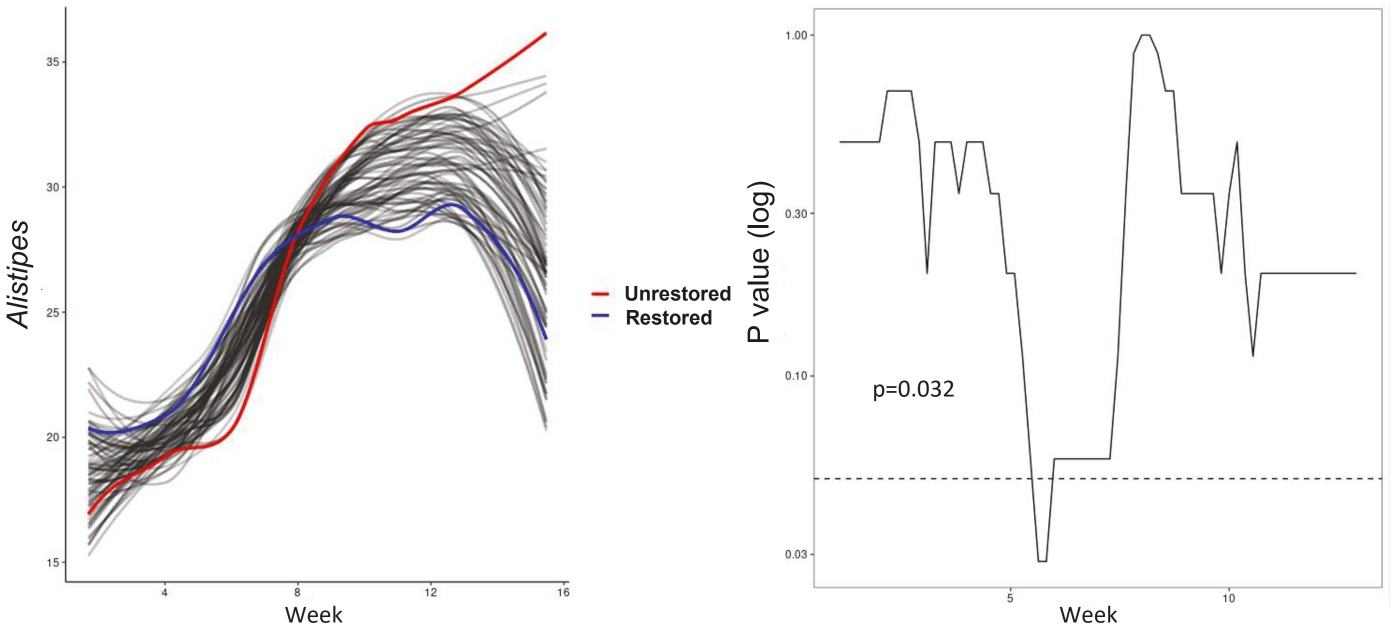

Supplementary Figure S1B

B)

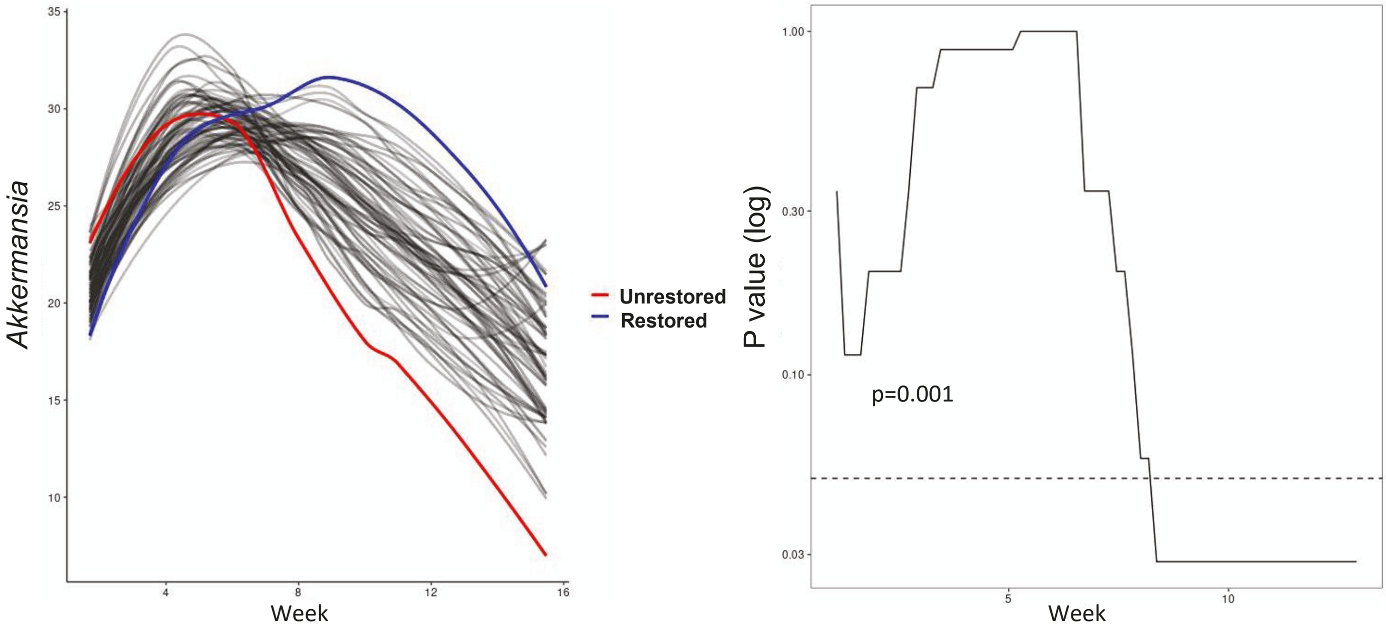

Supplementary Figure S1C

C)

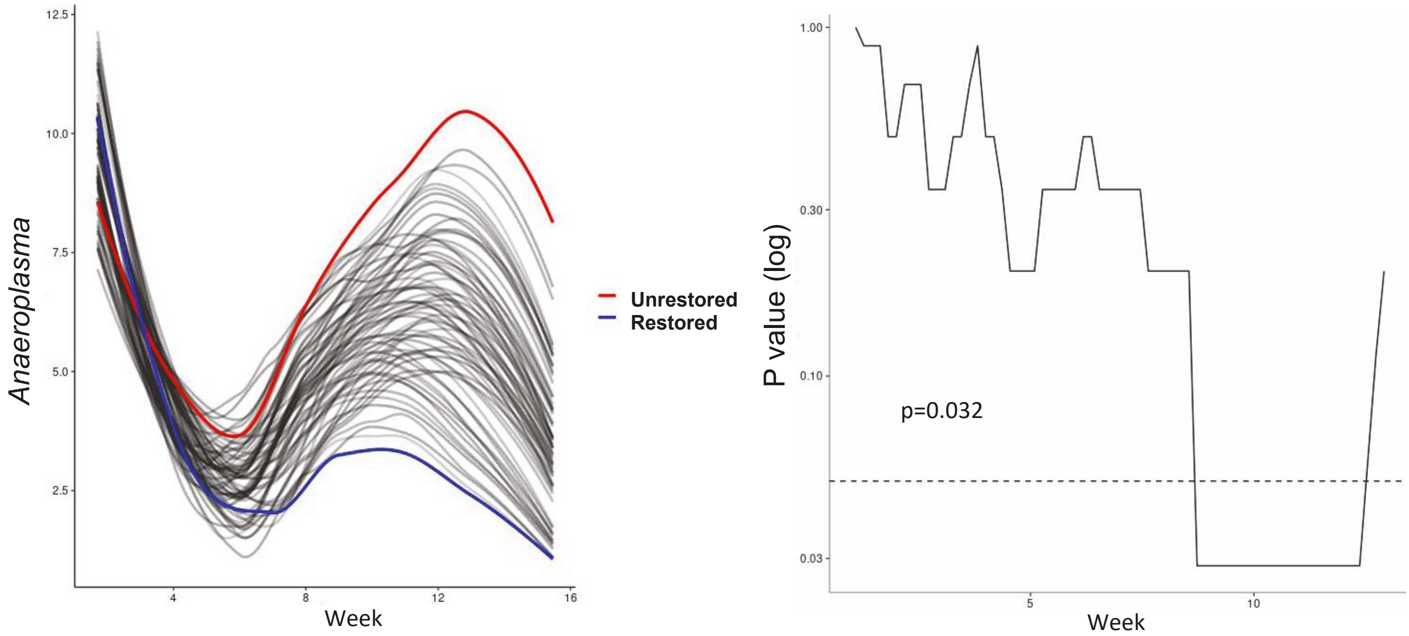

Supplementary Figure S1D

D)

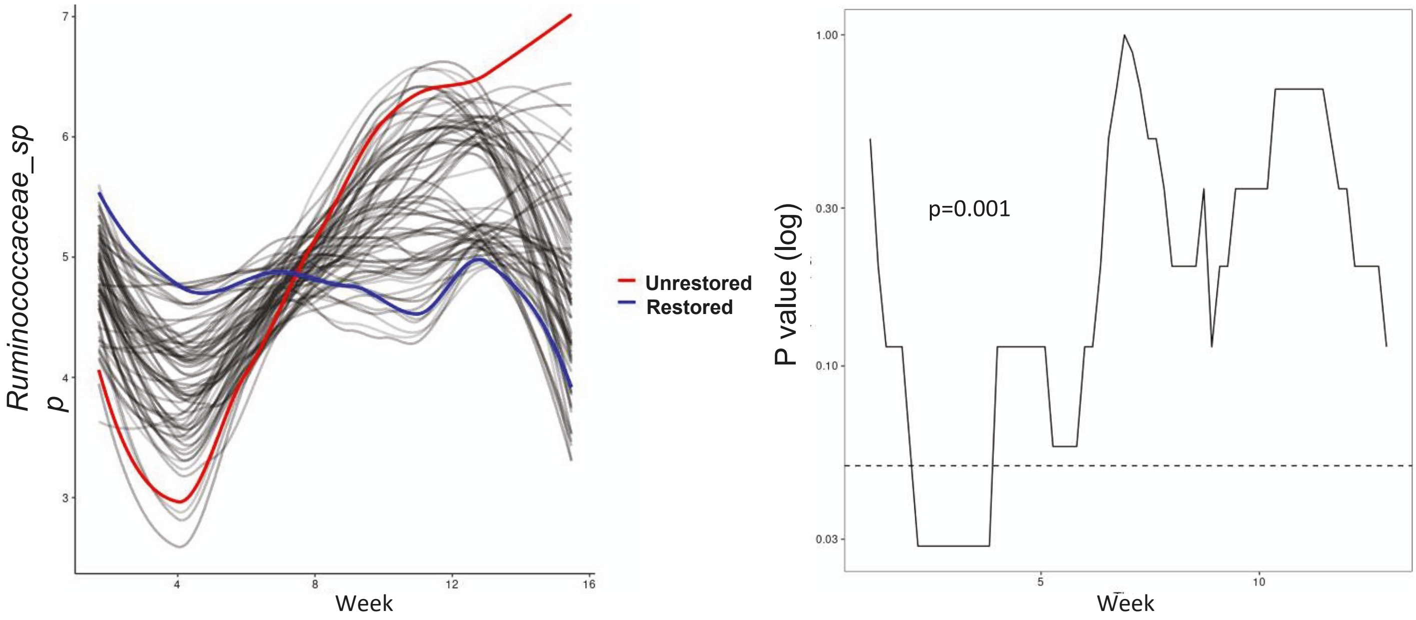

Supplementary Figure S1E

E)

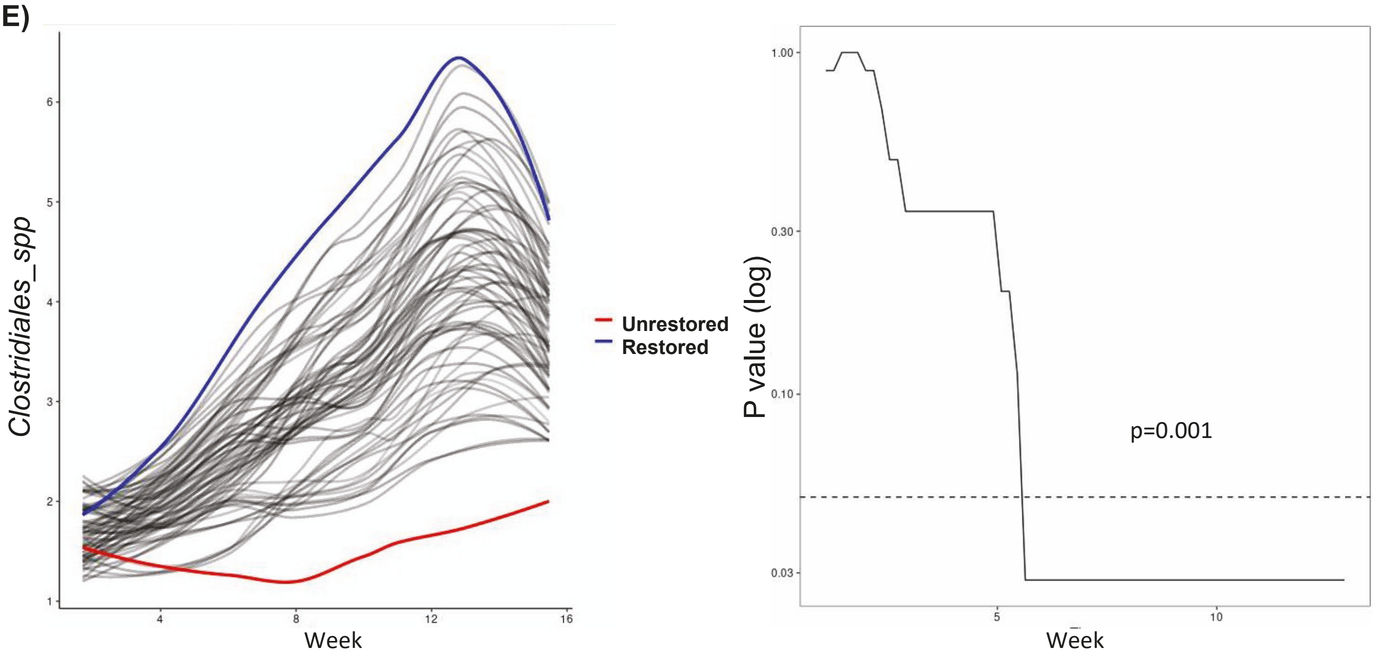

Supplementary Figure S1F

F)

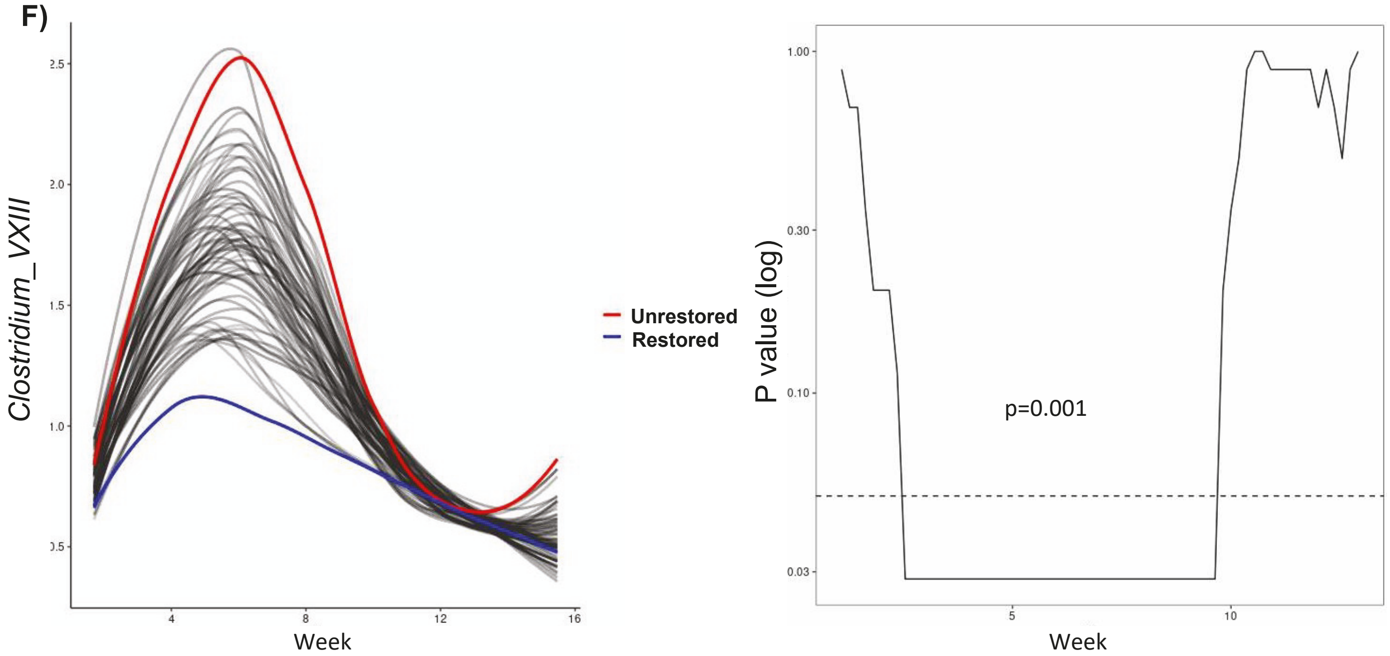

Supplementary Figure S1G

G)

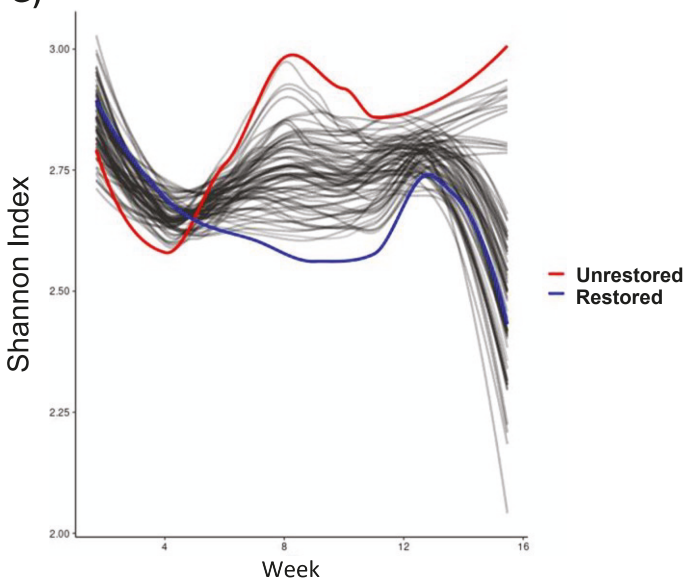

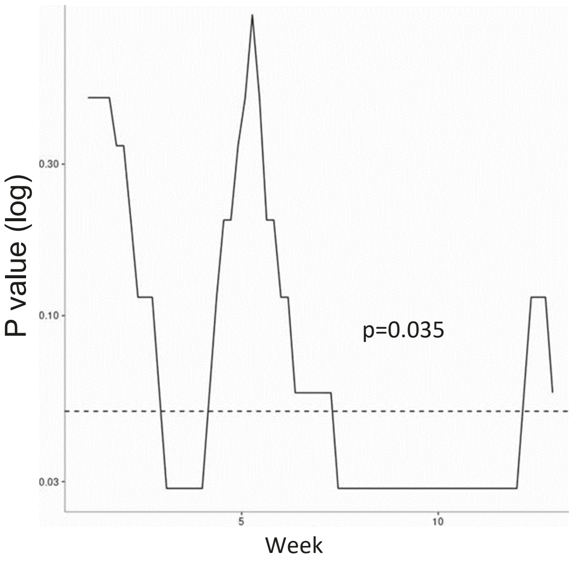

SF 1G

Supplementary Figure S2

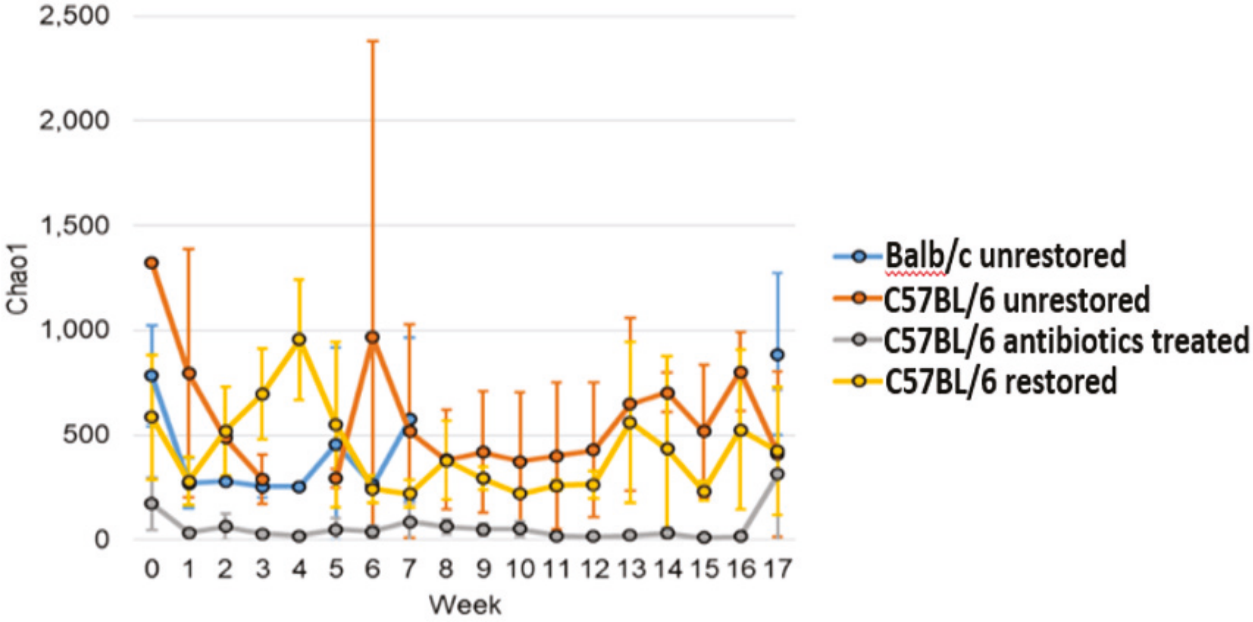

Supplementary Figure S3A

A)

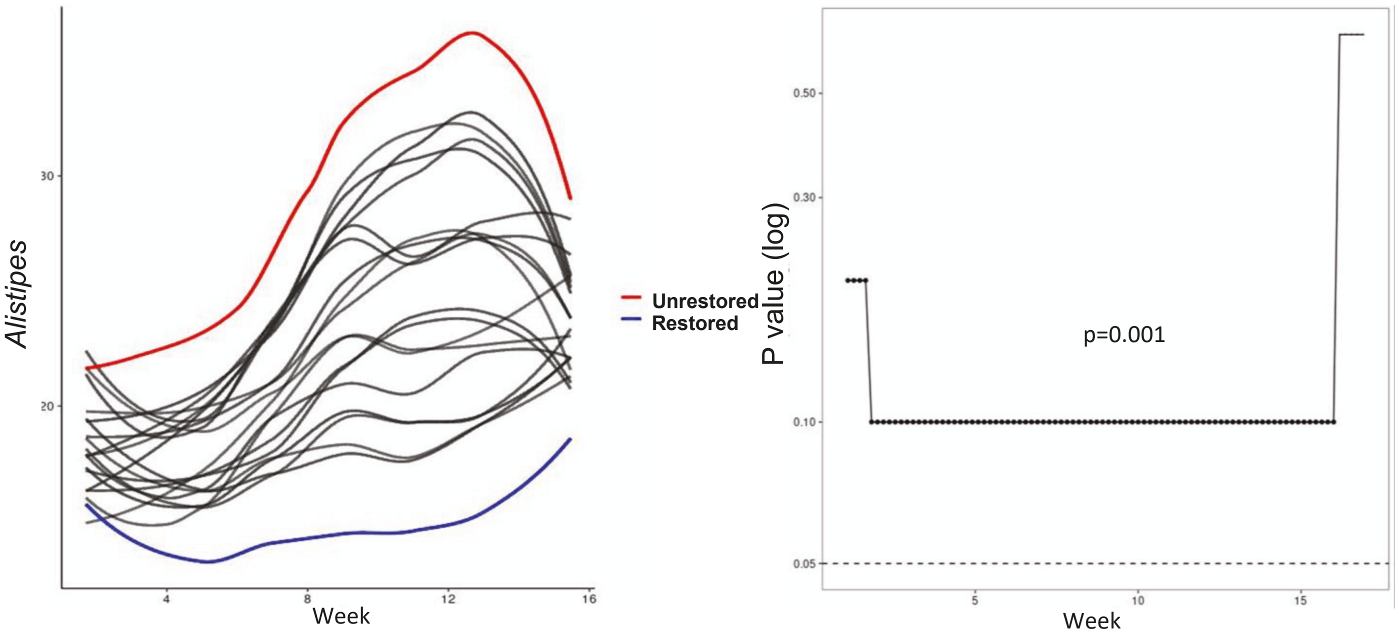

SF 3A

Supplementary Figure S3B

B)

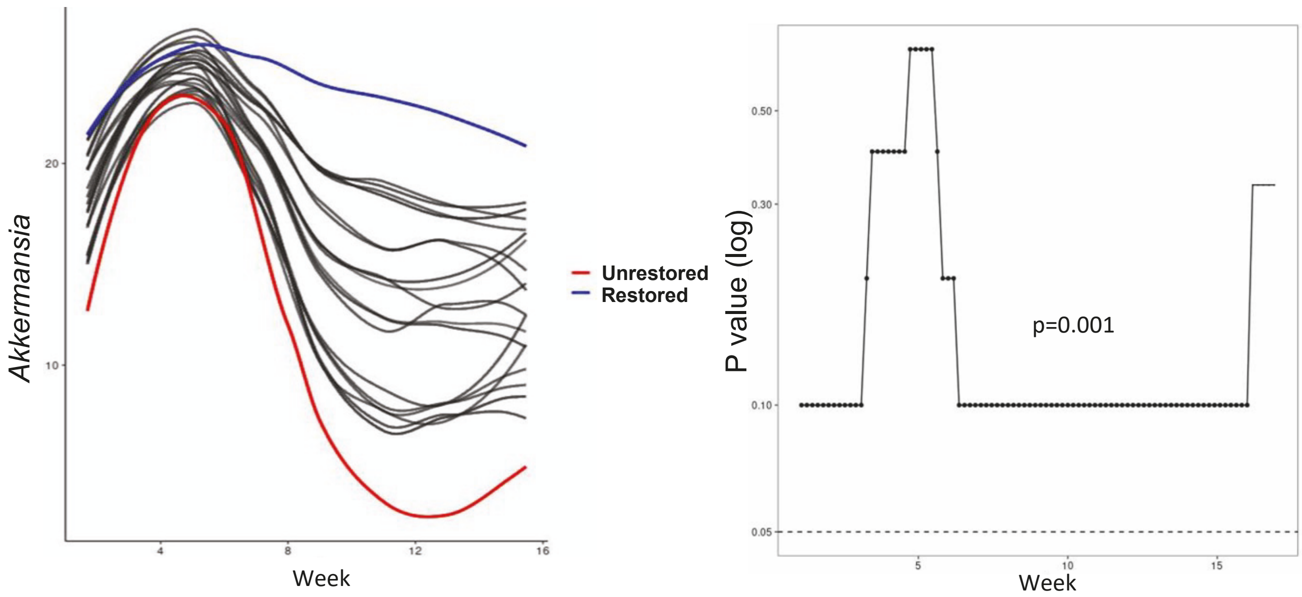

Supplementary Figure S3C

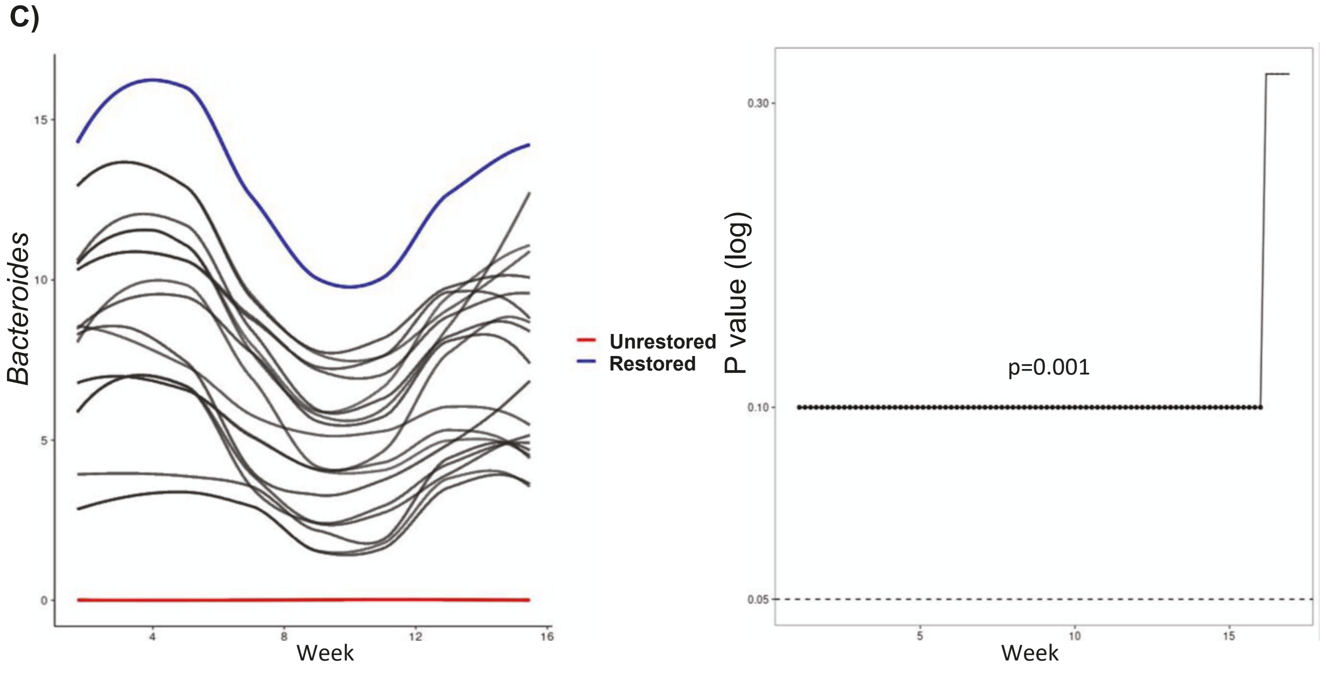

Supplementary Figure S3D

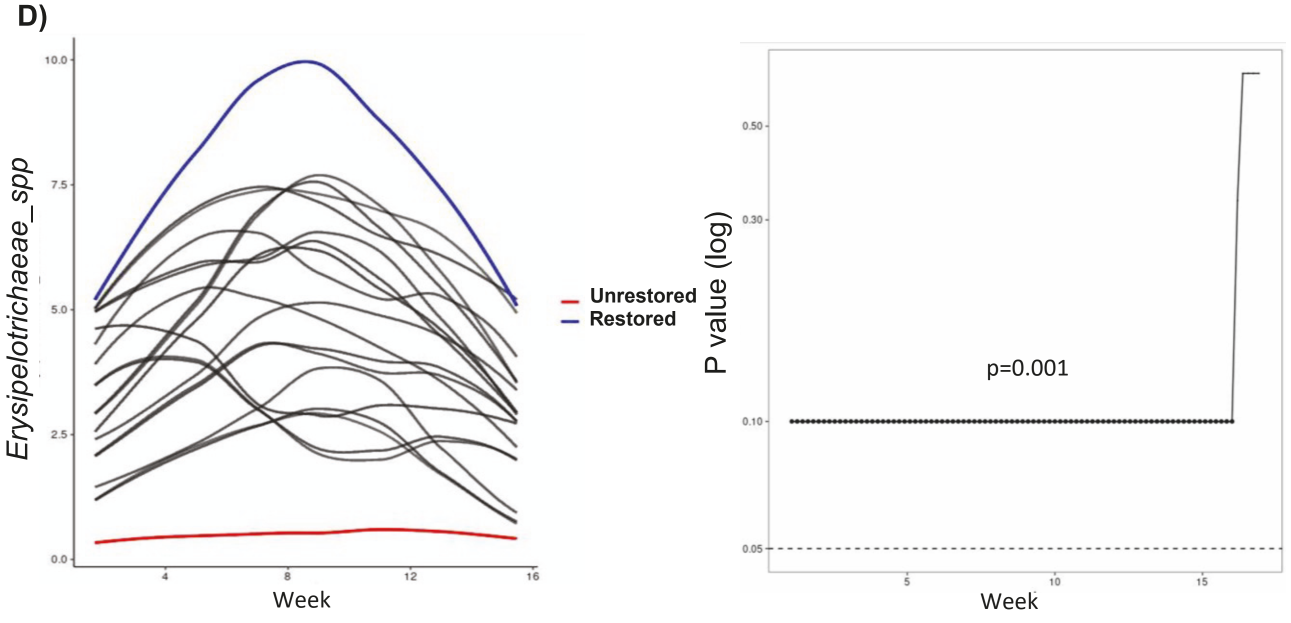

Supplementary Figure S3E

E)

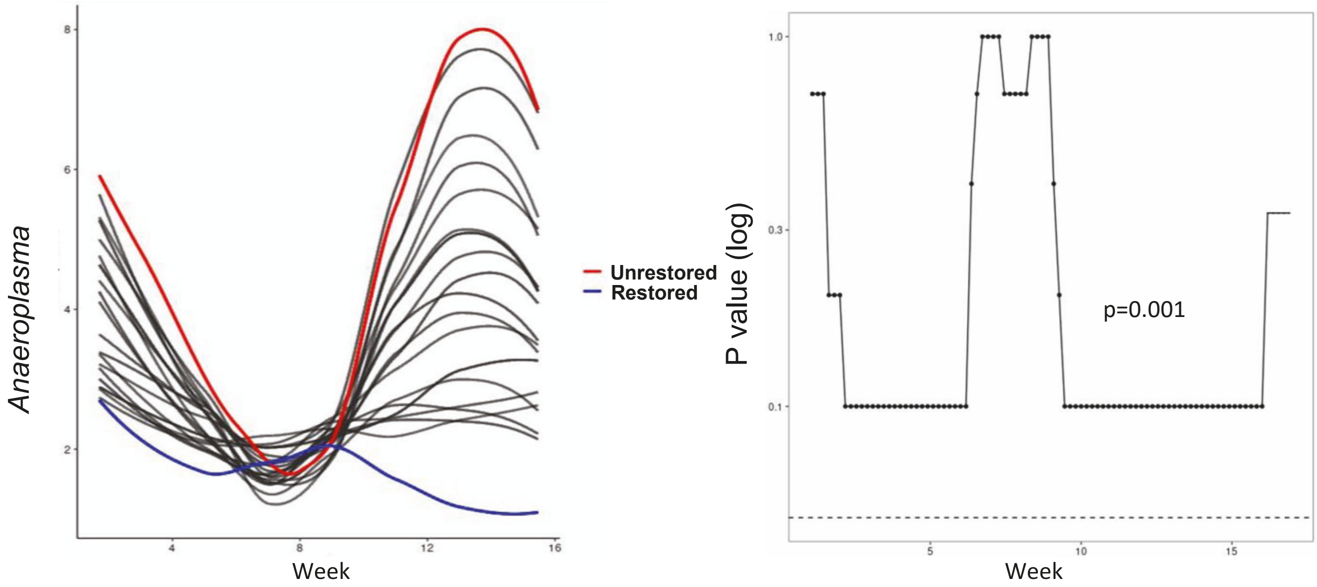

Supplement: Supplementary file 1 [file cancers-15-02260-s001.zip › cancers-2290927-supplementary.pdf]
